# Advance in the pathogenesis and treatment of Wilson disease

**DOI:** 10.1186/2047-9158-1-23

**Published:** 2012-11-27

**Authors:** Qin-Yun Dong, Zhi-Ying Wu

**Affiliations:** 1 Department of Neurology and Institute of Neurology, Huashan Hospital, Institutes of Brain Science and State Key Laboratory of Medical Neurobiology, Shanghai Medical College, Fudan University, 12 Wulumuqi Zhong Road, Shanghai, 200040, China

**Keywords:** Wilson disease, Copper, ATP7B, COMMD1, D-penicillamin, Trientine, Zinc, Ammonium tetrathiomolybdate, Liver transplantation

## Abstract

Wilson disease is an autosomal recessive disorder of copper metabolism. Diagnosis depends primarily on clinical features, biochemical parameters and the presence of the Kayser-Fleischer ring. Genetic analysis for mutations within *ATP7B* is a convincing diagnostic tool. The traditional treatment for WD includes chelation of excessive copper accumulation and reduction of copper intake. Medical therapy is effective but WD is not yet curable. Liver transplantation is especially helpful for patients who fail to respond to medical therapy or present with fulminant liver failure, although evaluation of its long-term effect are still in need.

## Introduction

Wilson disease (WD) is an autosomal recessive disorder, in which defective biliary excretion of copper results in its accumulation in liver, brain, kidney and cornea. The WD gene, *ATP7B*, is located on chromosome 13q14.3. It encodes a P-type ATPase, ATP7B. The incidence of WD is estimated to be 1 in 30,000 individuals, and the gene frequency is about 1 in 90 among many ethnic groups [1]. Diagnosis solely based on clinical manifestations and biochemical parameters lacks accuracy. Thus, a new diagnostic scoring system has been proposed [2], which includes the mutation analysis. Today, symptoms of WD can be effectively controlled with medical therapy and liver transplantation but it is still deemed incurable. Future efforts will likely involve gene therapy and other novel interventions. In this review, we will firstly review the pathogenesis of WD and secondly, discuss the advances in the treatment of WD.

### Pathogenesis

Copper is an essential trace element in the human body and a required component of many proteins. Excess copper causes oxidative damage to hepatocytes and allows spillage of free copper into the blood. It will overload other organs such as the brain, kidney and cornea, initiating toxic damage [1]. However, excessive cellular copper damages neuronal and metabolic functions [3], which is displayed by the wide spectrum of symptoms in WD. The defective gene, *ATP7B*, is responsible for this disorder. Despite intensive studies over the past decade, the mechanisms of how the defective ATP7B disrupts the copper excretion and regulations of ATP7B remain a myth.

### Copper homeostasis

The body’s essential copper requirement is about 1–2 mg per day. Dietary intake alone suffices the daily need. Copper is absorbed by the intestinal cells and stored with metallothionein in a non-toxic form. It is later delivered into the circulation by a copper transporter protein, ATP7A on the membrane of enterocytes [4]. The copper is then transported to the liver tagged with albumin, where it is uptaken by hepatocytes. Within these cells, the ATOX1 chaperone protein directs copper to its binding targets [5]. Some are bound to metallothionein, an intracellular chelator of harmful or excessive metal, for storage, and the rest is excreted into biliary canaliculi under the regulation of ATP7B.

The WD gene, *ATP7B*, encodes a putative copper-transporting P-type ATPase, ATP7B. ATP7B assumes double roles. It participates in the biliary excretion of copper and incorporates copper into nascent ceruloplasmin [6,7]. ATP7B has a trans-membrane organization (including 8 domains), an ATP-binding domain toward the carboxy terminus, and an amino tail comprised of 6 copper-binding units. It normally locates in the trans-Golgi network (TGN). ATP7B trafficking is regulated with its copper-translocation cycle, with cytosolic vesicular localization associated with the acyl-phosphate intermediate. Copper-chelation studies with some mutants clearly demonstrated a requirement for copper in ATP7B trafficking, suggesting the presence of an additional copper-binding site(s) within the protein [8].

Copper transportation capacity of ATP7B mutants is reduced or completely lost. One study using transient transfection and triple-label immunofluorescence microscopy shows that WD associated protein variation have different intracellular localizations compared with wild-type ATP7B protein [9]. This is later confirmed by Huster et al. in Sf9 cells. In addition, Huster et al. [10] points out that if the mutation causes ATP7B misfolding, it results in severe dysfunctions. Misfolded protein cannot exit from the ER, thus abolishing copper delivery. In addition, misfolding of ATP7B is correctable *in vitro* by pharmacological chaperones 4-phenylbutyrate and curcumin, though its application *in vivo* requires further elucidation [11].

ATP7B function is also impaired if the mutant has a dysfunctional ATP-binding domain. Using yeast as a model system, Hsi et al. [8] discovered that sequence variations in the ATP-binding domain of ATP7B affect copper transport. Not coincidentally, Huster et al. also discovered that ATP7B mutants would lose its copper delivery capacity with intact copper binding capacity if the mutations happen in ATP binding-domain or spatially-adjacent region [10].

### Copper toxicity

ATP7B dysfunction results in hepatic copper accumulation and subsequent overflow of copper to other organs like brain, kidney and cornea. Copper toxicity and mitochondrial dysfunction is closely related. The energy production of mitochondria is disrupted. In *ATP7B* knockout (*ATP7B*^−/−^) rats, Zischka et al. reports that liver mitochondria contain enlarged cristae, widened intermembrane spaces and a large amount of copper accumulation [12]. Detectable abnormalities, including impaired oxidative phosphorylation and biochemical signs of oxidative damage, come later. Respiratory chain complex I activity is increased in livers and brains of *ATP7B*^−/−^ mice, whereas complex II, III and IV activities are reduced [13]. Mitochondria have respiratory chain complex I as their major source of reactive oxygen stress production [14].

Besides, sterol metabolism is also severely disrupted [13]. Cardiolipin is an important component of mitochondrial inner membrane. The free-radical fragmentation of cardiolipin, phosphatidic acid (PA) and phosphatidylhydroxyacetone (PHA) are more abundant in the liver of *ATP7B*^−/−^ mice over 32 weeks old compared with control group at the same age [15]. It’s still not clear whether the copper-accumulation-induced oxidative stress leads to dysfunction of mitochondria or the copper accumulation in mitochondria leads to the production of oxidative stress. It’s probable that both mechanisms are equally important.

As a result of ROS injury and mitochondria dysfunction, apoptosis finally occurs. It is still unclear which pathways of apoptosis are responsible for cell loss in organ lesions of WD patients. Strand S et al. [16] discovered that copper (II) was an inducer of CD95 mediated apoptosis in livers of WD patients with fulminant liver failure. Addition of antioxidant and radical scavengers could inhibit apoptosis induced by copper [17]. The neuron degeneration is more complicated that apoptosis in the liver. Despite the detection of mitochondria autophagy, ROS injury alone can’t explain why dopaminergic neurons are more vulnerable to copper toxicity. Paris I et al. [18] pointed out that copper would form a complex with dopamine. In vitro, treatment with copper-dopamine complex would cause apoptosis of RCSN-3 cells (a cell line that expresses dopamine, norepinephrine and serotonin transporters). During the process, mitochondrial autophagy and oligonucleosomal DNA fragmentation occurred. The apoptosis is independent of caspase 3 pathway.

### Regulations of ATP7B

Some factors are believed to modulate the function of ATP7B, including Copper metabolism (Murr1) domain-containing protein 1 (COMMD1), clusterin, X-linked inhibitor of apoptosis (XIAP) and solute carrier family 33 (acetyl-CoA transporter) member 1 (SLC33A1).

COMMD1 is a protein responsible for canine copper metabolism disorder [19]. It facilitates the degradation of ATP7B. It specifically interacts with the amino-terminus of ATP7B. This interaction is independent of intracellular copper levels and copper chaperone antioxidant protein 1 (ATOX1) expression [20]. It is noticed that more COMMD1 binds to unstable, misfolded ATP7B [21]. Over-expression of COMMD1 decreases the amount of ATP7B expression whereas knockdown of COMMD1 increases it [21]. So far, sequencing analysis reports no evidence to implicate the COMMD1 protein in copper-storage disorders of undefined etiology in human beings [22].

Clusterin is recognized as an extracellular chaperone. It also facilitates ATP7B degradation. Its role closely relates with COMMD1. They can coexist in a complex. Clusterin knockdown and over-expression increases and decreases the levels of COMMD1 respectively. However, their mechanisms of regulating ATP7B levels are independent of each other. Whereas COMMD1 are sensitive to misfolded ATP7B, clusterin respond to oxidative stress [21].

X-linked inhibitor of apoptosis (XIAP), an anti-apoptotic protein, has recently been shown to regulate copper homeostasis. XIAP levels are greatly reduced in cells cultured under high copper conditions [23]. XIAP exerts its role in two different ways [24]: First, XIAP, when bounded with copper, will lead to its conformational changes, which results in increased susceptibility to apoptotic stimuli; second, XIAP promotes the degradation of COMMD1 by protoeasome. A novel mutation of its copper chaperone copper chaperone for superoxide dismutase (CCS) has been reported in a patient [25]. Although this CCS mutant doesn’t show impaired interaction with XIAP, further research is needed to determine whether CCS mutation is able to affect COMMD1 level by altering copper delivery to XIAP.

### Clinical management

#### Diet

One way to reduce copper absorption is to control the copper content of food. Patients should avoid having foods that contain high concentration of copper, such as chocolate, liver, nuts, mushrooms, and shellfish, and using the copper apparatus for food. Wu et al. have found low-copper diet and zinc are effective treatment for the presymptomatic WD patients [26].

#### Medical therap*y*

Traditional treatment of WD focuses on promotion of copper excretion and reduction of copper intake. Initial treatment for symptomatic patients with WD should include a chelating agent, D-penicillamine or trientine (bettered tolerated than D-penicillamine). Zinc is recommended by some experts as a first line therapy in neurological patients [27]. Maintenance therapy of presymptomatic patients or those with neurological symptoms usually requires a chelating agent or zinc [27]. Ammonium tetrathiomolybdate shows optimal effect in treating patients presented with neurological symptoms, though not yet commercially available [28,29]. The efficacy of copper excretion is measured by the levels of urinary copper.

##### D-penicillamine

D-penicillamine chelates copper inside the body. It mobilizes intracellular copper into the circulation and enhances urinary excretion of copper. McArdle et al. [30] found that D-penicillamine increases metallothionein mRNA levels without changing either the rate of copper uptake or the amount of copper within mouse hepatocytes. Metallothionein chelates the excessive copper to form a non-toxic combination [31]. On the contrary, Xu et al. [32] discovers that in Long-Evans Cinnamon (LEC) rats, an animal model of WD, excessive copper accumulation induces metallothionein in hepatocytes whereas D-penicillamine reduces the level of both metallothionein-l mRNA and metallothionein protein.

The initial dose of penicillamine is 750–1500 mg per day in two to four divided doses for adults. Dosing in children is 20 mg/kg/day to the nearest 250 mg, divided in two or three divided doses [27]. The treatment is best taken 1 hour before or 2 hours after food. Absorption is estimated to be only 50% if it is taken with a meal. The use of lower initial doses, 125–250 mg per day, increasing over a few weeks, can enhance tolerance to the agent. Pyridoxine (vitamin B6) is added routinely to the treatment regimen in a dosage of 20–50 mg daily, as its deficiency is associated with neurological worsening induced by D-penicillamine [33], which is irreversible in some cases.

The use of D-penicillamine remains controversial for decades due to its adverse effects. Someone suggests that D-penicillamine should not be used as initial therapy in WD [34,35]. Apart from neurological worsening, they include early reactions such as fever, rash, lymphadenopathy or late reactions such as bone marrow and renal toxicity [36]. Newly reported unfavorable effects consist of ANCA-vasculitis in WD [37] and dermatology toxicity, such as progeric changes in the skin, such as pemphigous or pemphigoid lesions [38]. Severe adverse effects necessitate discontinuation of D-penicillamine and change of regimen in 20-30% of patients [39,40].

The reasons for the neurological deterioration of the D-penicillamine are still unknown. Depletion of pyridoxine may be one of them [33]. Radical therapies that drastically lower serum copper level are found to induce partial status epilepticus as well as severe deterioration of neurological symptoms in one patient, whose conditions improved shortly after discontinuing D-penicillamine and having a high copper diet [41]. Increased free copper concentrations are found in the serum and brain, and declined protein-bound copper concentrations are found in the brain of toxic milk mice during D-penicillamine administration [42]. Immunoflurescence staining shows intense staining of ATP7A in the choroid plexus but rare ATP7A and copper transporter 1 (CTR1) on the blood–brain-barrier, which suggests that D-penicillamine mobilizes free copper from the brain parenchyma rather than from the blood.

During administration of D-penicillamine, periodic clinical, hematological, biochemical and routine urinary parameters are monitored weekly for 1 month, then monthly for 6 months and at 6-month intervals thereafter. It’s notable that cessation of penicillamine without replacement treatment causes rapid progression to fatal fulminant hepatitis [43].

##### Trientine

Trientine has a polyamine structure, which chelates copper by the formation of stable complexes with the four constituent nitrogens in a planar ring. It is debatable whether the two agents mobilize different pools of copper. The initial dose is 900–2700 mg per day in two to three divided doses. Maintenance therapy is 900–1500 mg per day. As in the case of penicillamine, trientine should be given orally 1 hour before or 2 hours after food [27].

Trientine is a less potent copper remover than D-penicillamine with its toxic profile similar to that of D-penicillamine, although side effects are less frequent and generally milder [44]. It is believed that neurological deteriorations are less frequent in trientine than in D-penicillamine [45]. However, Weiss et al. [46,47] reported a less frequent neurological deterioration in patients treated with D-penicillamine than with trientine while the extent of improvement was similar. Weiss et al. [47] reported that neurological deteriorations happened to patients in all treatment regimen including D-penicillamine, trientine, zinc and combination therapy, and the P values were not significant.

Evidence grows for the effectiveness of trientine. Askari et al. [48] studied 9 adults with severe liver disease identified over a 10 year period. They received initial treatment with trientine (1000 mg/day) and zinc (150 mg/day). Only one patient had hepatic encephalopathy. One patient developed mild neurological symptoms so the patient was given ammonium tetrathiomolybdate and zinc after 2 weeks of the original treatment. In the eight patients receiving trientine and zinc, the combination was given for at least 4 months and then maintenance zinc treatment was used. Over the first 12 months of treatment, prothrombin time and raised bilirubin and albumin concentrations returned to normal, and ascites disappeared. Benefit was maintained over 12 months to 14 years of follow-up. Taylor et al. [40] provides evidence that trientine is as efficacious as penicillamine with a lower side effect profile in pediatric patients. However, trientine is not yet commercially available in China.

##### Zinc

Zinc induces intestinal metallothionein, which preferentially binds to copper within the duodenal enterocytes**.** Thus, copper absorption into the circulation is reduced, and copper is lost when the enterocytes are shed during normal cell turnover. Continuing copper losses in combination with reduced absorption lead to a negative copper balance. Furthermore zinc can induce copper-binding metallothionein in hepatocytes, thereby reducing the damaging effects of free copper.

Zinc has been used successfully in asymptomatic or presymptomatic patients. Wu et al. [26] have identified 17 presymptomatic patients with WD by genetic analysis and prophylactic treatment of 14 patients with zinc over 3 to 5 years resulted in lowered level of urinary copper. None of the patients developed clinical symptoms of WD or adverse effects of zinc therapy by the end of the study period. In contrast, 3 patients who refused treatment had symptomatic progression. Brewer et al. [49] conclude that zinc is effective as a sole therapy and that it has low toxicity based on data from the long-term follow-up of maintenance zinc treatment of 141 symptomatic and presymptomatic patients with WD. It’s the same for children and pregnant women, although more data should be acquired. In patients with severe hepatic disease, maintenance therapy with zinc was effective after an initial period of treatment with trientine and zinc [48].

When the patients are given zinc monotherapy, liver functions should be regularly monitored. Among patients with hepatic manifestations, non-response to zinc monotherapy is not uncommon. This is not due to noncompliance. If the level of AST, ALT and γ-GT remains in a high level after the initiation of the zinc therapy, it often suggests a poor response and a change in therapy should be considered as soon as possible [50].

Patients discontinue zinc therapy mostly due to gastrointestinal discomfort. It is more common with zinc sulfate than zinc acetate. During a long-term follow-up by Bruha et al., with a median follow-up of 12 years, no adverse events were reported with zinc acetate [50].

##### Ammonium tetrathiomolybdate

Tetrathiomolybdate has a unique mechanism of action. Its four sulfur groups allow it to form a stable tripartite complex with copper and protein [51,52]. Ammonium tetrathiomolybdate is well-absorbed with or without food. It forms a complex with copper in the food. When it is given away from food, it forms a complex with available copper and albumin so that it can’t be up-taken or utilized for intracellular process [29].

Although not yet commercially available, its high efficacy and rare neurological deterioration compared with trientine makes it a potential cure for WD patients with neurological symptoms [53]. One out of 25 (4%) patients treated with tetrathiomolybdate while 6 of 23 (26%) patients treated with trientine showed neurological deterioration during an 8-week study [45]. Unlike trientine, tetrathiomolybdate stably lowers the serum “free copper” level, which may explain uncommon neurological deterioration. Its recommended dose is 120 mg daily as 20 mg with meals and 20 mg between meals for 2 weeks, and then 60 mg daily as 10 mg 3 times daily with meals and 10 mg 3 times daily between meals [53].

##### Symptomatic treatment

Despite the generally well response to chelation therapy with zinc, additional treatments to control the disturbing symptoms are still necessary for some patients. Neurological symptoms are usually more refractory than hepatic damages. Neurological symptoms can be categorized into parkinsonism, dysarthria, dystonia, tremor, pseudosclerosis [54]. Parkinsonism can be treated with L-dopa, dopamine agonists [54] and anticholinergics(trihexyphenidyl) [55]. Essential tremor-like tremor is treated with beta-blocker (propranolol) or barbituate (primidone) [55]. Dystonia can be treated with trihexyphenidyl, baclofen, valium or dopamine antagonist (tiapride) and botulinem injection [56,57]. Proxysmal dystonic movement can be treated with oxcarbazepine [56]. Gabapentine can be used when dealing with penicillamine-induced dystonia when other options fail [58]. However, there are only a small number of case reports regarding symptomatic treatments and the conclusions are inconsistent.

##### Experience from China

In China, most Departments of Neurology treat WD patients according to WD guideline. A few neurologists choose traditional Chinese medicine (TCM) and decoppering agents other than D-penicillamine and trientine. Besides, aggressive decoppering therapies involving intravenous 2, 3-dimercaptopropane-1-sulfonate (DMPS) will also be applied. Unfortunately, there are only a limited number of reports regarding this aspect. Aggressive decoppering therapies are theoretically dangerous for patients because rapid mobilization and depletion of deposited copper are associated with neurological worsening. However, according to the aggressive therapy reported by Yang et al. [59], 84.85% of the patients showed improvement and 11.11% of the patients were symptom free based on Goldstein activity of daily life scale. However, the account of adverse events was not given.

TCM is mostly used in combination with decoppering agents. The most commonly used TCM agents include Gandou Decoction, Gandou Tablets, and oxymatrine. The choice of TCM agents is empirical and lacking consensus among neurologists. So far, there is no strong evidence to recommend TCM in treating WD due to the methodical limitations of these clinical researches. Most researches did not provide information on how the random allocation was generated and concealed. Based on a systematic review of 9 randomized controlled trials [60], TCM therapy are generally associated with better response and fewer adverse events. However, 8 out of the 9 randomized control trials mentioned above lacked blinding methods and none of them reported dropout. As a result, the optimistic outcomes of TCM therapy are questionable.

#### Liver transplantation

There are some reports of successful management of acute liver disease by medical therapy [61]. However, if medical therapy fails to suppress the progression of the disease, orthotopic liver transplantation (OLT) is the only alternative treatment. It is indicated for WD patients with acute liver failure when the revised King’s score is 11 or higher and patients with decompensated cirrhosis that’s unresponsive to chelation therapy [27]. Liver transplantations of these individuals can be achieved by a cadaveric donor or living donor transplant, even if the donor is a heterozygous carrier [27].

OLT generally gives excellent results. The one year survival rate and 5 year survival rate in pediatric patients are 90.1% and 89% in comparison with a survival of 88.3% and 86% for adults in a multi-center observational study over 20 years [62]. The OLT also corrects the underlying renal disorder [63]. However, survival is only one aspect of patients’ well-being. Neurological complications can be quite common. The immunosuppressive agents would leave the patients vulnerable to infections. Lack of compliance in the immunosuppressive agents would lead to acute hepatic failure [64]. Long-term follow-up would be in need to assess the efficacy of OLT.

Liver transplantation is not recommended for patients with neurological and psychiatric symptoms [65]. However, some patients choose to undergo transplantation for their neurological impairment and some patients with liver damage show neurological improvement after the surgery [66-70]. Otherwise, there are risks associated with the procedure of liver transplantation and the following immunosuppressive therapy [71,72]. According to Enrol et al. [73], 10/17 of the pediatric patients who were free from neurological symptoms before the operations have as many as 16 episodes afterwards. The author largely attributes these complications to the use of immunosuppressive agent tacrolimus and cyclosporine instead of a manifestation of latent neurological symptoms. The serum chemistry panel suggested that those patients didn’t have metabolic disorders although hepatic encephalopathy prior to the surgery was a possible risk factor for post-operational neurological complications. MRI also revealed posterior leukoencephalopathy syndrome in 2 patients with seizure but EEG didn’t reveal any epileptiform abnormalities. These complications tended to be short-lived and resolved promptly after discontinuing or reducing the dose of immunosuppressive agents [73]. Other observations show, although the mild neurological symptoms may improve after OLT, concomitant severe or moderate neurological and particularly neuropsychiatric symptoms are negative prognostic factors for post-OLT survival [74].

There are few absolute contraindications for liver transplantation. Patients with poor cardiac and pulmonary functions, namely patients with severe hypoxia or right arterial pressure greater than 60 mm Hg rarely survive surgery and the perioperative recovery period. Before transplantation is attempted, patients with extrahepatic malignancies other than squamous cell skin carcinoma should be postponed for at least 2 years after the curative therapy is completed. Significant psychiatric or neurological disorders and ongoing destructive behavior caused by substance abuse must be under effective medical control to make sure that the patient can be compliant after transplantation. Absence of a viable splanchnic venous inflow system is the most common surgical contraindication to liver transplantation. If the entire portal venous system is occluded, the transplantation is rarely successful. Plus, uncontrolled systemic infection is apparently an contraindication to high-dose immunosuppressive therapy [65,75].

Are the decoppering treatment and the low copper diet necessary after the transplant? There is no report yet. Long-term results of liver transplantation are still in lack of large cohort studies. The development of better prognostication for neurological progression or improvement of WD by MRI findings or other clinical or biochemical variables will help improve our abilities to make treatment choices.

#### Pregnancy

Female WD patients can become pregnant if her copper status optimized prior to pregnancy. Although there is some concern over the teratogenicity of D-penicillamine, withdrawing treatment is more risky than continuing it. Among 161 reported cases of pregnancies in 83 women with WD (one of them had successful *in vitro* fertilization) treated with D-penicillamine during pregnancy showed 122 births with 119 normal newborns [27].

Low risk of teratogenicity is also true for treatment with trientine [76] or zinc [77]. Breast feeding under chelation therapy is not recommended, although there are reports that children breast fed by mothers on D-penicillamine show no abnormalities [78]. It is recommended to lower D-penicillamine during the first trimester and put patients on the lowered dosage for all trimesters with continued monitoring. Others recommend administering chelators at a minimal dose, i.e. 300–600 mg/day in the last trimester to avoid insufficient copper supply to the fetus or insufficient wound healing after Cesarean section or episiotomy [79].

#### The future

Patients faced with a lifelong need for medication and physicians faced with the results of non-adherence to therapy are the two main arguments. Genetic therapy and hepatocyte transplantation represent future curative treatments for WD, along with currently available liver transplantation. Therapeutic replacement of organs with healthy cells requires disease-specific strategies. As copper toxicosis due to ATP7B deficiency in WD produces significant liver injury, disease-specific study of transplanted cell proliferation will offer insights into cell and gene therapy mechanisms. Studies on animal model have showed that cell therapy will correct genetic disorders characterized by organ damage, but suitable mechanisms for inducing transplanted cell proliferation will be critical for therapeutic success [80]. However, both cell and liver transplants need immunosuppression to maintain grafted cells. Future use of stem-cells, ex-vivo modification of cells by gene therapy, or better means of inducing immune tolerance may obviate the difficulty of immunosuppresion and provide a cure for WD by cell transplantation.

## Abbreviations

WD: Wilson Disease; COMMD1: Copper metabolism (Murr1) domain-containing protein 1; XIAP: X-linked inhibitor of apoptosis; SLC33A1: Solute carrier family 33 (acetyl-CoA transporter), member 1; ATOX1: Antioxidant protein 1; CCS: Copper chaperone for superoxide dismutase; TCM: Traditional Chinese Medicine; OLT: Orthotopic liver transplantation.

## Competing interests

Both authors declare that they have no competing interest.

## Authors' contributions

DQY performed the literature search and drafted the manuscript. WZY reviewed relevant articles, critically revised the manuscript, and made ongoing recommendations regarding necessary additions or changes to the article. All authors read and approved the final manuscript.
